# Is Coronal Restoration a Predictor of Posttreatment Apical Periodontitis?

**DOI:** 10.1055/s-0041-1735909

**Published:** 2021-11-09

**Authors:** Romana Persic Bukmir, Ema Paljevic, Jelena Vidas, Irena Glazar, Sonja Pezelj-Ribaric, Ivana Brekalo Prso

**Affiliations:** 1 Department of Endodontics and Restorative Dentistry, Faculty of Dental Medicine, University of Rijeka, Rijeka, Croatia; 2 Department of Oral Medicine and Periodontology, Faculty of Dental Medicine, University of Rijeka, Rijeka, Croatia; 3 Department of Dental Medicine, Faculty of Dental Medicine and Health, Josip Juraj Strossmayer University of Osijek, Osijek, Croatia

**Keywords:** dental, digital periapical radiograph, apical periodontitis, permanent coronal restoration, root canal treatment

## Abstract

**Objectives** To investigate the posttreatment apical periodontitis (AP) in endodontically treated teeth through a multivariate approach and to analyze the relative importance of quality and type of coronal restoration as predictors of periapical disease.

**Materials and Methods** The present study sample was drawn within 2-year period from the 1,072 consecutive patients older than 18 years, first time attending the Dental Clinic of the Clinical Hospital Center Rijeka, Croatia. A total of 1,350 endodontically treated teeth were included in the study. For each tooth, the periapical status, root filling quality, intracanal post, separated file presence, marginal bone loss, and quality and type of coronal restoration were recorded.

**Statistical Analysis** Chi-square tests were used to analyze the variations in the periapical status, quality of root canal filling, and quality of coronal restoration in different tooth types. The effect of explanatory variables on periapical status was explored using univariate and multivariate logistic regression models. The outcome variable was set as the presence versus absence of AP in the tooth.

**Results** Multivariate logistic regression analysis revealed statistically significant associations and increased risk for AP presence in molars (odds ratio [OR] = 2.15; *p* < 0.001), teeth positioned in mandible (OR = 1.49; *p* = 0.007), teeth with short length of root filling (OR = 4.08; *p* < 0.001), overfilled teeth (OR = 2.99; *p* = 0.001), and teeth with inadequate density of root filling (OR = 4.14; *p* < 0.001). Considering variables related to coronal restoration, neither coronal restoration type nor quality was found to be predictive for posttreatment AP. Merely, the presence of intracanal post significantly increased the odds of AP presence (OR = 1.57; *p* = 0.009).

**Conclusion** The results of the present study did not indicate that type or quality of coronal restoration may be predictors of posttreatment AP. Periapical disease was significantly associated with molars, mandibular teeth, substandard quality of root fillings, and intracanal post presence.

## Introduction

The importance of natural teeth preservation cannot be overemphasized. In fact, when given options are extraction and root canal treatment, endodontic treatment should be treatment of choice as it can provide the best health and cosmetic result. 1 The intention of nonsurgical root canal treatment is to provide conditions for the healing of periapical tissues by eliminating infection from the root canal system and to keep the tooth's function in the oral environment. 2 3

Cross-sectional studies conducted in different European, American, and Asian populations reported that endodontically treated teeth are prevalent among adults, ranging from 1.5 to 21% out of all examined teeth. 4 5 6 7 Posttreatment disease, a phrase suggested by Friedman, describes the persistence of apical periodontitis (AP) in endodontically treated teeth. 8 Studies conducted at dental schools, where endodontic treatment was performed or supervised by an endodontic specialist reported a success rate to be more than 90%. 8 9 10 11 On the other hand, the studies reflecting a more realistic outcome of endodontic treatments in the general populations reported the prevalence of AP in endodontically treated teeth to range from 25 to more than 65%. 4 12 Therefore, posttreatment AP constitutes a significant health problem in many populations and attempts have been made to identify prognostic factors for this disease. Yet, further investigations are required to improve the outcomes of root canal treatment and benefits related to the oral health of the population.

Postendodontic restoration of the tooth crown is an essential factor in the reinstitution of the tooth function and the prevention of coronal leakage. Studies have attempted to disclose the role of the coronal restoration type and quality in outcome of root canal treatment. A recent study reported that neither the type nor the material of the restoration was significant for periapical status of endodontically treated teeth if the quality of the restoration was acceptable. 13 Conversely, a study conducted in Sweden revealed that besides the quality of the root filling, restoration type may also be predictive of AP in root-filled teeth. 14 *In vivo* studies investigating the impact of coronal restoration and root fillings on periapical status reported inconsistent results. Ray and Trope, and Kirkevang et al reported that quality of coronal restorations had a significantly greater impact on periapical status than the quality of root canal filling. 15 16 Several studies identified both the coronal restoration and root canal treatment quality as a predictor of periapical status, 17 18 19 while some studies implicated a lesser impact of the coronal restoration quality on the outcome of the endodontic treatment in comparison to the quality of root canal treatment. 20 21

The hypothesis for the present study was that the periapical condition of endodontically treated teeth significantly varies with regard to the type and quality of permanent coronal restorations. This study aimed to explore posttreatment AP in endodontically treated teeth through a multivariate approach and to analyze the relative importance of type and quality of coronal restoration as predictors of periapical disease.

## Materials and Methods

This study protocol was approved by the Ethical Committee of the Clinical Hospital Center Rijeka (003-05/13-01/03). Study sample was drawn within 2 years from the 1,072 consecutive patients older than 18 years, who attended the Dental Clinic of the Clinical Hospital Center Rijeka, Croatia for the first time.

Participants were excluded if they declined to participate in the study, had seven or less remaining teeth, received endodontic therapy within previous 2 years, and were unwilling or unable to attend the radiographic diagnostics. To be enrolled in the survey, the patient's chart had to comprise panoramic radiograph. After applying these criteria, the sample consisted of 597 participants who agreed to take part by signing an informed consent. The study was conducted in accordance with the World Medical Association Declaration of Helsinki principles.

Endodontically treated teeth were identified from panoramic radiographs. Teeth were recorded as endodontically treated if radiopaque material was visible in pulp chamber and/or root canal(s). Teeth with temporary or missing restorations were excluded from further analysis. Digital periapical radiographs of all permanently restored endodontically treated teeth were taken with paralleling technique using X-ray unit (Trophy Elitys; Trophy Radiologie, Marne-la-Vallee, France) and intraoral sensor (One; Owandy Radiology, Roslyn, New York, United States). The applied exposure parameters were 60 kV, 7 mA, and 0.25 seconds. Images were analyzed on a 19-inch liquid crystal monitor (P1914S; Dell, Austin, Texas, United States; resolution: 1.280 × 1.024 32-bit color; graphic card: HD Graphic; Intel, Santa Clara, California, United States). For each tooth, the periapical status, root filling quality, intracanal post and separated file presence, marginal bone loss, and quality and type of coronal restoration were recorded. All criteria are listed in Table 1 .

**Table 1:** Variables scored in root-filled teeth

Variables	Codes
Tooth type	0 = Anterior 1 = Premolar 2 = Molar
Arch type	0 = Maxilla 1 = Mandible
Apical periodontitis 22	0 = Absent (PAI score = 1, 2)
	1 = Present (PAI score = 3, 4, 5)
Length of root filling 5	0 = Adequate (ending 0–2 mm short of the radiographic apex)
	1 = Short (ending more than 2 mm from the radiographic apex)
	2 = Long (extruding beyond the radiographic apex)
Density of root filling 5	0 = Adequate (uniform radiodensity and adaptation of the filling to the root canal walls)
	1 = Inadequate (visible canal space laterally along the filling or voids within the filling mass, or identifiable untreated canal)
Quality of root filling (combined criteria for length and density of root filling) a	0 = Adequate (ending 0–2 mm from radiographic apex, no voids present)
	1 = Inadequate (ending > 2 mm short of, or extending beyond the radiographic apex, presence of voids, or untreated canal)
Intracanal post 17	0 = Absent (absence of a metal or fiber post in the root canal)
	1 = Present (presence of a metal or fiber post in the root canal)
Separated file	0 = Absent
	1 = Present
Marginal bone loss 13	0 = No marginal bone loss (≤1/3 of the root length)
	1 = Marginal bone loss (>1/3 of the root length)
Type of coronal restoration 14	0 = 1–3 surfaces amalgam
	1 = 4–5 surfaces amalgam
	2 = 1–3 surfaces composite
	3 = 4–5 surface composite
	4 = Inlay
	5 = Crown
Quality of coronal restoration (clinically and radiographically) 14 23 24	0 = Adequate (no defective restoration margin, no signs of recurrent caries)
	1 = Inadequate (defective restoration margin and/or presence of recurrent caries)
Combined quality of root filling and coronal restoration a	0 = Adequate root filling/adequate coronal restoration
	1 = Adequate root filling/inadequate coronal restoration
	2 = Inadequate root filling/adequate coronal restoration
	3 = Inadequate root filling/inadequate coronal restoration

Abbreviation: PAI, periapical index.

a Variables used only in univariate analysis.

**Fig. 1:**
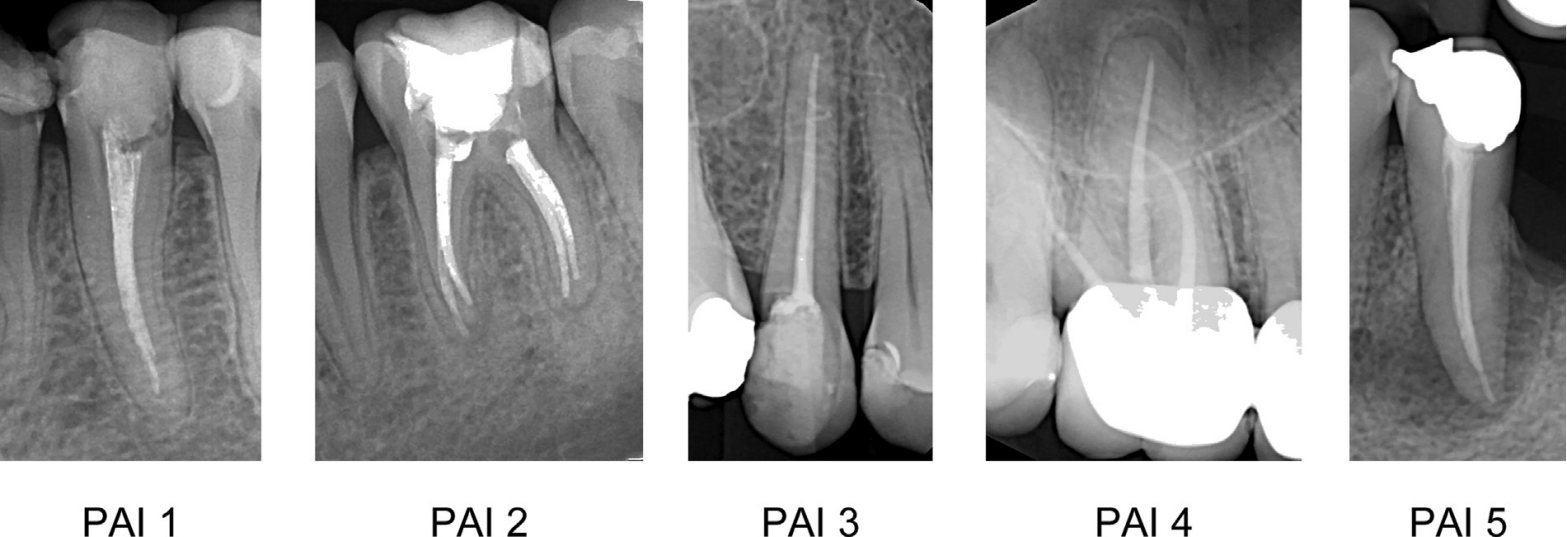
Periapical radiographs of endodontically treated teeth originating from investigated material representing each of the five periapical index (PAI) scores. Absence of apical periodontitis: PAI = 1 and PAI = 2. Presence of apical periodontitis: PAI = 3, PAI = 4, and PAI = 5.

Analysis of the endodontic variables and marginal bone loss was accomplished using periapical radiographs. The AP presence was assessed utilizing the periapical index (PAI) system. 22 Visual references for the full-scale PAI were applied to determine the periapical condition of each tooth. In multirooted teeth, the highest PAI value of all roots was used to define the periapical status. PAI scores were dichotomized, and AP was recorded as absent (PAI scores 1 and 2) or present (PAI scores 3, 4, and 5; Fig. 1 ). The root filling quality was scored with respect to length and density according to previously reported criteria. 5 Marginal bone loss was estimated as less or more than one-third of the root length. 13

The quality of coronal restoration was assessed clinically and radiographically with respect to marginal integrity of restoration and recurrent caries presence. Modified U.S. Public Health Service/Ryge criteria for marginal integrity and recurrent caries were used for the clinical evaluation of coronal restoration as reported by Merdad et al. 23 Marginal integrity was radiographically assessed as adequate (radiographically sealed) or inadequate (signs of open margins or overhangs) according to previously described criteria. 24 Recurrent caries was recorded as absent or present in case of clearly visible decrease in mineral content of a proximal tooth surface contiguous with a restoration. 14

All data were recorded by one observer. Observer's agreement to PAI scores from 100 reference radiographs resulted in Cohen's kappa value of 0.70. 22 Calibrations for assessment of dental caries, coronal restoration quality, quality of root canal filing, and marginal bone loss were performed according to the World Health Organization recommendations. 25 Intraobserver agreement was analyzed by double scoring of the 30 randomly selected study participants' clinical and radiographic surveys. Kappa values for clinical and radiographic diagnosis of recurrent caries were 0.85 and 0.92, respectively, while clinical and radiographic assessment of marginal integrity yielded kappa values of 0.81 and 0.85, respectively. Intraobserver kappa values were 0.75 for PAI, 0.80 and 0.85 for length and density of the root canal filling, and 0.89 for marginal bone loss.

### Statistical Analysis

Statistical calculations were performed using MedCalc statistical software (MedCalc Software Ltd., Belgium) at level of statistical significance *p* < 0.05. Lilliefors' test was used to test data for distribution normality. As the data did not distribute normally, median and interquartile range were used to report central tendency and dispersion. Mann–Whitney's *U* test was used to investigate the differences between the groups regarding the continuous variables. Chi-square tests were used to analyze the significant variations in periapical status, quality of root canal filling, and quality of coronal restoration according to the tooth type. Univariate logistic regression was applied to explore the effect of diverse variables on periapical status. The variables with significant associations in univariate model were included in multivariate logistic regression analysis to identify significant predictors of the outcome variable. The outcome variable was set as the presence versus absence of AP in the tooth.

## Results

The majority of sample consisted of women (68.2%). The average age of participant was 34 years (interquartile range 24–46). No significant differences between male and female participants were determined regarding age (Mann–Whitney's *U* test; *p* = 0.534), median number of root-filled teeth (Mann–Whitney's *U* test; *p* = 0.387), or median number of root-filled teeth with AP (Mann–Whitney's *U* test; *p* = 0.181). Root-filled teeth were found in 448 (75.0%) participants, while 334 (54.3%) participants had one or more root-filled teeth with radiological signs of AP. No significant difference was demonstrated between male and female participants considering the prevalence of endodontic treatment (χ ^2^ = 0.15; *p* = 0.697) and posttreatment AP (χ ^2^ = 1.27; *p* = 0.260).

Of the 1,350 endodontically treated teeth investigated in the present study, the distribution included 386 (28.6%) anterior, 430 (31.9%) premolar, and 534 molar teeth (39.6%). AP was present in 634 teeth (47%). Most of examined teeth had inadequate quality of root filling (67.6%) and coronal restoration (60.1%).

Significant differences in the root filling quality (χ ^2^ = 41.83; *p* < 0.001) and the coronal restoration quality (χ ^2^ = 15.25; *p* < 0.001) regarding the tooth type were observed. Molars had significantly higher proportion of inadequate root fillings than anterior (76.6 vs. 56.6%; *p* < 0.001) and premolar teeth (76.6 vs. 66.3%; *p* < 0.001). Also, poor quality of coronal restoration was significantly more frequent in molar than anterior (66.5 vs. 57.3%; *p* < 0.005) and premolar teeth (66.5 vs. 54.9%; *p* < 0.001). Furthermore, the periapical status significantly differed regarding the tooth type (χ ^2^ = 64.29; *p* < 0.001). Only 215 molars (40.3%) were designated as periapically healthy, while 255 (66.1%) anterior and 246 (57.2%) premolar teeth had no radiological signs of AP. When compared with anterior and premolar teeth, molars had significantly higher proportion of periapical disease (both *p* < 0.001; Table 2 ).

**Table 2:** Distribution of teeth according to the tooth type with differences in the quality of the root filling, quality of coronal restoration, and periapical status

Tooth type	No. of teeth	Quality of root filling			Quality of coronal restorations			Periapical status		
		Adequate *N* (%)	Inadequate *N* (%)		Adequate *N* (%)	Inadequate *N* (%)		AP absence	AP presence	
Anterior	386	168 (43.5)	218 (56.5)	b	165 (42.7)	221 (57.3)	c	255 (66.1)	131 (33.9)	b
Premolar	430	145 (33.7)	285 (66.3)	b	194 (45.1)	236 (54.9)	c	246 (57.2)	184 (42.8)	b
Molar	534	125 (23.4)	409 (76.6)	b	179 (33.5)	355 (66.5)	b	215 (40.3)	319 (59.7)	b
Total	1350	438 (32.4)	912 (67.6)		538 (39.9)	812 (60.1)		716 (53.0)	634 (47.0)	
Statistics		χ ^2^ = 41.83 *p* < 0.001 a			χ ^2^ = 15.25 *p* < 0.001 a			χ ^2^ = 64.29 *p* < 0.001 a		

Abbreviation: AP, apical periodontitis.

a Statistically significant (chi-square test).

b Significant difference versus both other tooth types.

c Significant difference versus molars.

Table 3 demonstrates the distribution of root-filled teeth presenting with and without AP according to independent variables and their associations with periapical status. An increased risk for AP presence was demonstrated for premolars (odds ratio [OR] = 1.46; 95% confidence interval [CI]: 1.10–1.94; *p* = 0.010) and molars (OR = 2.89; 95% CI: 2.20–3.79; *p* < 0.001). Mandibular teeth were more frequently affected than maxillary teeth constituting 56.1% of AP findings with an increased odds of AP presence (OR = 1.73; 95% CI: 1.30–2.17; *p* < 0.001). Significant variations in periapical status regarding quality of root filling were found. Based on combined criteria for length and density of the root canal filing, the overall quality of the root canal filling was rated as adequate in only 438 (32.4%) of the examined teeth. Of these teeth, 385 (87.9%) had no radiologic signs of AP. AP was the least frequent in teeth with adequate length of root canal filing (17.1%) compared with teeth with short length of root canal filling (69.8%) and teeth with root canal filling extended beyond the apex (40.0%). An increased risk for AP was demonstrated in underfilled (OR = 11.19; 95% CI: 8.51–17.71; *p* < 0.001) and overfilled teeth (OR = 3.23; 95% CI: 1.93–5.39; *p* < 0.001). It was observed that 56.7% of teeth had inadequate density of root filling with AP prevalence of 68%. This group demonstrated an increased risk for AP presence (OR = 8.86; 95% CI: 6.90–11.44; *p* < 0.001). Intracanal post was detected in 276 teeth (20.4%). The proportion of AP in teeth restored with intracanal post was 52.9%, demonstrating higher odds for AP than in teeth without the post (OR = 1.35; 95% CI: 1.03–1.76; *p* = 0.030).

**Table 3:** Univariate logistic regression for associations of diverse variables and apical periodontitis in root-filled teeth

Variables	Total	AP absent *N* (%)	AP present *N* (%)	OR	95% CI	*p* -Value
Tooth type						
Anterior	386	255 (66.1)	131 (33.9)	1	Reference	
Premolar	430	246 (57.2)	184 (42.8)	1.46	1.10–1.94	0.010 a
Molar	534	215 (40.3)	319 (59.7)	2.89	2.20–3.79	<0.001 a
Arch type						
Maxilla	906	521 (57.5)	385 (42.5)	1	Reference	
Mandible	444	195 (43.9)	249 (56.1)	1.73	1.3–2.17	<0.001 a
Length of root filling						
Adequate	543	450 (82.9)	93 (17.1)	1	Reference	
Short	732	221 (30.2)	511 (69.8)	11.19	8.51–14.71	<0.001 a
Long	75	45 (60.0)	30 (40.0)	3.23	1.93–5.39	<0.001 a
Density of root filling						
Adequate	584	471 (80.7)	113 (19.3)	1	Reference	
Inadequate	766	245 (32.0)	521 (68.0)	8.86	6.90–11.44	<0.001 a
Quality of root filling						
Adequate	438	385 (87.9)	53 (12.1)	1	Reference	
Inadequate	912	331 (36.3)	581 (63.7)	12.75	9.28–17.51	<0.001 a
Intracanal post						
Absent	1,074	586 (54.6)	488 (45.4)	1	Reference	
Present	276	130 (47.1)	146 (52.9)	1.35	1.03–1.76	0.030 a
Separated file						
Absent	1,311	701 (53.5)	610 (46.5)	1	Reference	
Present	39	15 (38.5)	24 (61.5)	1.84	0.96–3.54	0.068
Marginal bone loss						
≤1/3 root length	963	506 (52.5)	457 (47.5)	1	Reference	
> 1/3 root length	387	210 (54.3)	177 (45.7)	0.933	0.74–1.18	0.567
Type of coronal restoration						
1–3 surface amalgam	48	21 (43.8)	27 (56.2)	1	Reference	
4–5 surface amalgam	67	29 (43.3)	38 (56.7)	1.02	0.48–2.15	0.960
1–3 surface composite	472	259 (54.9)	213 (45.1)	0.64	0.35–1.16	0.143
4–5 surface composite	312	171 (54.8)	141 (45.2)	0.64	0.35–1.18	0.155
Inlay	13	8 (61.5)	5 (38.5)	0.49	0.14–1.70	0.260
Crown	438	228 (52.1)	210 (47.9)	0.72	0.39–1.31	0.276
Quality of coronal restoration						
Adequate	538	330 (61.3)	208 (38.66)	1	Reference	
Inadequate	812	386 (47.5)	426 (52.5)	1.75	1.40–2.19	<0.001 a

Abbreviations: AP, apical periodontitis; CI, confidence interval; OR, odds ratio.

a Statistically significant.

Considering high prevalence of teeth with inadequate quality of coronal restoration, a significant association with periapical disease presence was found (OR = 1.75; 95% CI: 1.40–2.19; *p* < 0.001; Table 3 ). Variables separated file, marginal bone loss, or type of coronal restoration was not significantly associated with the presence of periapical disease.

Association of outcome variable (AP) and combined data for root filling quality and coronal restoration quality were analyzed by means of univariate logistic regression ( Table 4 ). Teeth with both adequate quality of root filling and restoration exhibited the lowest prevalence of AP (12.2%) and were used as a reference category. In case of adequate root filling and inadequate restoration, AP was observed in 12.2% of teeth; however, the risk for AP presence was not increased in this group. Conversely, combination of inadequate quality of root filling and adequate quality of restoration was significantly associated with AP (OR = 10.35; 95% CI: 6.59–16.24; *p* < 0.001). The highest association with AP presence was observed in case of inadequate quality of both analyzed parameters (OR = 13.76; 95% CI: 9.02–21.00; *p* < 0.001). The highest prevalence of AP of 65.6% occurred in this group ( Table 4 ).

**Table 4:** Univariate logistic regression for association of combined quality of root canal filling and coronal restoration with apical periodontitis

Covariate	No. of teeth	AP absence *N* (%)	AP presence *N* (%)	OR	95% CI	*p* -Value
Adequate root filling/adequate restoration	238	209 (87.8)	29 (12.2)	1	Reference	
Adequate root filling/inadequate restoration	196	172 (87.8)	24 (12.2)	1.01	0.57–1.79	0.985
Inadequate root filling/adequate restoration	302	124 (41.1)	178 (58.9)	10.35	6.59–16.24	<0.001 a
Inadequate root filling/inadequate restoration	614	211 (34.4)	403 (65.6)	13.77	9.02–21.00	<0.001 a

Abbreviations: AP, apical periodontitis; CI, confidence interval; OR, odds ratio.

a Statistically significant.

The results of multivariate logistic regression analysis are presented in Table 5 . Only variables with significant associations in univariate analysis (except overall quality of root filling) were included in multivariate model. Five variables maintained significant associations with the AP presence: tooth type, dental arch, length and density of the root canal filling, and intracanal post. Regarding the tooth type and dental arch, the OR for AP presence was 2.15 times higher in molars (OR = 2.15; 95% CI: 1.51–3.06; *p* < 0.001) than in anterior teeth, while compared with maxillary dental arch, teeth positioned in mandible had 1.49 times increased odds of AP presence (OR = 1.49; 95% CI: 1.11–1.99; *p* = 0.007). When compared with teeth with adequate length of the root filling, the OR for the presence of AP was 4.08 times higher if the length of the root filling was short (OR = 4.08; 95% CI: 2.93–5.69; *p* < 0.001) and 2.99 times higher in the case of the overfilling (OR = 2.99; 95% CI: 1.71–5.24; *p* = 0.001). Teeth with inadequate density of root filling had 4.14 times increased odds of AP presence (OR = 4.14; 95% CI: 3.01–5.69; *p* < 0.001) than those with adequate density. The presence of intracanal post increased the risk for tooth having AP 1.57 times (OR = 1.57; 95% CI: 1.12–2.22; *p* = 0.009; Table 5 ).

**Table 5:** Multivariate logistic regression for association of diverse variables and apical periodontitis in root-filled teeth

Variables	No. of teeth	Odds ratio	95% CI	*p* -Value
Tooth type				
Anterior	386	1		
Premolar	430	1.28	0.91–1.81	0.162
Molar	534	2.15	1.51–3.06	<0.001 a
Arch type				
Maxilla	906	1		
Mandible	444	1.49	1.11–1.99	0.007 a
Length of root filling				
Adequate	543	1		
Short	732	4.08	2.93–5.69	<0.001 a
Long	75	2.99	1.71–5.24	<0.001 a
Density of root filling				
Adequate	584	1		
Inadequate	766	4.14	3.01–5.69	<0.001 a
Intracanal post				
Absent	1074	1		
Present	276	1.57	1.12–2.22	0.009 a
Quality of coronal restoration				
Adequate	538	1		
Inadequate	812	1.29	0.98–1.70	0.070

Abbreviations: AP, apical periodontitis; CI, confidence interval; OR, odds ratio.

a Statistically significant.

## Discussion

The prevalence of posttreatment AP in endodontically treated teeth in the current study was 47%, which was rather high but comparable to the range reported by other cross-sectional studies conducted among different populations. 4 5 6 7 17 Approximately 68% of teeth had poor quality of root filling, while 60% had substandard quality of coronal restoration. Molar teeth yielded the highest prevalence of AP (59.7%). This is not surprising since almost 77% of them had inadequate root canal filling quality, and the quality of coronal restoration was categorized as substandard in more than 66% molars. Difficulties encountered in treatment of molar teeth include inherent anatomical complexity and distal position. These limitations may be the explanations why most molars have substandard quality of root filling, yet the reasons for poor quality of coronal restorations remain unclear. Our results are consistent with most of the other studies reporting the highest prevalence of AP in endodontically treated molars. 5 6 17

Most of the analyzed teeth had inadequate quality of root filling (63.7%). As expected, teeth with both adequate length and density of root filling exhibited significantly less posttreatment AP than teeth with inadequate features of root filling. Interestingly, while underfilled teeth were most frequently affected with AP constituting almost 70% of the findings, teeth with root filling extending beyond the apex had significantly better periapical condition with AP prevalence of 40%. A recent systematic analysis reported a significantly increased risk of nonhealing outcome in case of endodontic material extrusion. 26 Others report that sealer extrusion did not compromise the success of endodontic treatment. 27 28 Given that currently used root filling materials vary regarding biocompatibility, physical and chemical properties, further studies are necessary to clarify this issue.

Instrument separation is an unpleasant complication occurring during the preparation of root canal and may affect the endodontic treatment outcome. 29 The present study revealed no significant difference in the periapical status regarding the presence of separated file. Treatment outcome is likely to be influenced by the stage of canal preparation and control of microbial infection when instrument separation occurs. Instrument fracture occurring in early stages of canal preparation would compromise disinfection and obturation of the root canal and significantly influence tooth prognosis. 30 However, it seems that tooth prognosis is not significantly affected by instrument separation in case the treatment is performed under high technical standards. 31

A study evaluating periodontal status of teeth indicated for undergoing endodontic treatment reported the rate of the periodontally compromised teeth to be almost 20%. 32 The present results demonstrated marginal bone loss of more than one-third of the root length in almost 29% of teeth. Though previous studies reported an association between marginal bone level and AP, 13 33 our results did not demonstrate any significant variations in periapical condition regarding the marginal bone loss.

Combined data for the root filling and coronal restoration quality were analyzed. Same prevalence of AP was observed in teeth with both adequate root filling and adequate coronal restoration, and in teeth with adequate root filling and inadequate restoration (both 12.2%). Significantly, worse outcome was observed in case of inadequate root filling quality, regardless of the coronal restoration quality. Congruent with previous investigation, our results may imply that the root filling quality may be more significant prognostic variable for posttreatment AP than quality of coronal restoration. 34

In the present study, the type of coronal restoration was not correlated with AP presence. The quality of coronal restoration demonstrated significant association with periapical status in univariate analysis; however, this association did not remain significant when multivariate analysis was applied. This may be due to the influence of other significant variables in the multivariate logistic regression model. Therefore, the hypothesis that the periapical status of endodontically treated teeth significantly varies with regard to the type and quality of permanent coronal restorations could not be verified. As in previous studies, the present results confirmed the strong association between the technical quality of root filling and AP presence. 13 14 17 The mandibular teeth had a higher risk for AP, and molars likewise presented significantly increased risk for AP presence. A significant association between the intracanal post presence and periapical disease was observed. It was argued that this may be due to root canal contamination during restoration procedure. 8 Other studies reported no difference between teeth with or without a post. 17 18 19

Considering the variety of factors that can influence the endodontic treatment failure, posttreatment AP should be investigated through multivariate models as they reflect better approximation to reality and allow for estimation of the relative importance of each predictor variable. However, it should be kept in mind that due to cross-sectional study design, there are variables that could not have been controlled and yet might have impacted the results. Endodontic treatment of teeth is a highly demanding procedure, and besides the obturation quality, its success also depends on mechanical instrumentation and disinfection effectiveness. Personal skills of operator are likely to influence the outcome of root canal treatment. This survey included participants who attended the dental clinic for the first time. According to the data reported by participants, general practitioners were providers of endodontic treatment in teeth included in the present study. However, no information regarding the endodontic treatment protocol were available. Time of postendodontic coronal restoration placement and proper aseptic technique application are also aspects that could not be assessed in this study.

Only participants who had endodontic treatment performed more than 2 years ago were recruited in the present study. A comprehensive study investigating factors affecting nonsurgical root canal treatment reported that more than 95% of periapical lesions completely healed within 2 years following root canal treatment. 35 Therefore, recruiting the participants who had endodontic treatment performed more than 2 years prior to this study seemed a reasonable time interval to avoid possible overestimation of AP presence in case of the periapical radiolucency that represents a stage of healing.

The limitations of conventional radiography, such as periapical and panoramic radiographs, are well established. 36 37 The main disadvantages are a two-dimensional nature of generated image with anatomical noise, masking the area of interest, and geometric distortion. 37 Due to high sensitivity in detection of periapical lesion and ability to provide high detail of the root canal system, the use of small field of views cone beam computed tomography (CBCT) in diagnosis and management of endodontic disease is increasing. When compared with CBCT, both panoramic and periapical radiographs correctly identify AP only in advanced stages, and therefore, the prevalence of AP may be underestimated. 38 However, the potential benefits of CBCT must be balanced with comparatively higher levels of risk from radiation exposure. The presence of metallic restorations (e.g., amalgam restorations, metal posts, crowns, and implants) or even gutta-percha can cause significant radiographic artifact, sufficient to compromise details of root canal anatomy and relevant pathosis. 37 Furthermore, it was demonstrated that the diagnostic accuracy of CBCT in detection of AP was high for non-root-filled teeth, while the diagnosis of AP on root-filled teeth was less accurate. 39 Despite the excellent accuracy of CBCT in AP diagnostics, it is still recommended to limit its use in endodontics on cases when conventional radiographic techniques do not provide sufficient information for confident diagnosis and treatment planning. 40 Considering the advantages and limitations inherent in each technique, and the cross-sectional design of the present study involving a large number of participants, the authors opted for periapical radiographs for AP diagnosis.

## Conclusion

Within the limitations of the present survey, it may be concluded that even though the coronal restoration quality is important in outcome of endodontic treatment, it seems that it is not a decisive factor. Multivariate analysis revealed that molars, mandibular teeth, short and long root fillings, inadequate density of root filling, and intracanal post are most important predictors for posttreatment periapical disease.
